# A Dynamical Systems Model for Combinatorial Cancer Therapy Enhances Oncolytic Adenovirus Efficacy by MEK-Inhibition

**DOI:** 10.1371/journal.pcbi.1001085

**Published:** 2011-02-17

**Authors:** Neda Bagheri, Marisa Shiina, Douglas A. Lauffenburger, W. Michael Korn

**Affiliations:** 1 Department of Biological Engineering, Massachusetts Institute of Technology, Cambridge, Massachusetts, United States of America; 2 Division of Gastroenterology and Helen Diller Family Comprehensive Cancer Center, University of California San Francisco, San Francisco, California, United States of America; University of Illinois at Urbana-Champaign, United States of America

## Abstract

Oncolytic adenoviruses, such as ONYX-015, have been tested in clinical trials for currently untreatable tumors, but have yet to demonstrate adequate therapeutic efficacy. The extent to which viruses infect targeted cells determines the efficacy of this approach but many tumors down-regulate the Coxsackievirus and Adenovirus Receptor (CAR), rendering them less susceptible to infection. Disrupting MAPK pathway signaling by pharmacological inhibition of MEK up-regulates CAR expression, offering possible enhanced adenovirus infection. MEK inhibition, however, interferes with adenovirus replication due to resulting G1-phase cell cycle arrest. Therefore, enhanced efficacy will depend on treatment protocols that productively balance these competing effects. Predictive understanding of how to attain and enhance therapeutic efficacy of combinatorial treatment is difficult since the effects of MEK inhibitors, in conjunction with adenovirus/cell interactions, are complex nonlinear dynamic processes. We investigated combinatorial treatment strategies using a mathematical model that predicts the impact of MEK inhibition on tumor cell proliferation, ONYX-015 infection, and oncolysis. Specifically, we fit a nonlinear differential equation system to dedicated experimental data and analyzed the resulting simulations for favorable treatment strategies. Simulations predicted enhanced combinatorial therapy when both treatments were applied simultaneously; we successfully validated these predictions in an ensuing explicit test study. Further analysis revealed that a CAR-independent mechanism may be responsible for amplified virus production and cell death. We conclude that integrated computational and experimental analysis of combinatorial therapy provides a useful means to identify treatment/infection protocols that yield clinically significant oncolysis. Enhanced oncolytic therapy has the potential to dramatically improve non-surgical cancer treatment, especially in locally advanced or metastatic cases where treatment options remain limited.

## Author Summary

Novel cancer treatment strategies are urgently needed since currently available non-surgical methods for most solid malignancies have limited impact on survival rates. We used conditionally replicating adenoviruses as cancer-fighting agents since they are designed to target and lyse cells with specific aberrations, leaving healthy cells undamaged. Highly malignant cells, however, down-regulate the adenovirus receptor, impairing infection and subsequent cell death. We demonstrated that disruption of the MEK pathway (which is frequently activated in cancer) up-regulated this receptor, resulting in enhanced adenovirus entry. Although receptor expression was restored, disruption of signaling interfered with adenovirus replication due to cell cycle arrest, presenting an opposing trade-off. We developed a dynamical systems model to characterize the response of cancer cells to oncolytic adenovirus infection and drug treatment, providing a means to enhance therapeutic efficacy of combination treatment strategies. Our simulations predicted improved therapeutic efficacy when drug treatment and infection occurred simultaneously. We successfully validated predictions and found that a CAR-independent mechanism may be responsible for regulating adenovirus production and cell death. This work demonstrates the utility of modeling for accurate prediction and optimization of combinatorial treatment strategies, serving as a paradigm for improved design of anti-cancer combination therapies.

## Introduction

Therapeutic options for most patients with locally advanced or metastatic cancer are limited. Surgery is often not an option for these patients because the cancer has diffusely spread, and currently available non-surgical treatments for most solid malignancies have insufficient impact on survival rates. Therefore, novel treatment strategies that incorporate the molecular composition of individual tumors are urgently needed. Conditionally replicating oncolytic adenoviruses are designed to target and lyse cells with specific aberrations, showing promise as a new non-surgical treatment strategy [1], [2]. The selective replication of viruses in cancer cells leads to destruction of infected cells by virus-mediated lysis. Consequently, the released viral progenies spread through the tumor mass by infecting neighboring cancer cells, resulting in self-perpetuating cycles of infection, replication, and oncolysis [3], [4]. As this approach relies on viral replication, the virus can, theoretically, self-amplify and spread in the tumor from an initial infection of only a few cells.

ONYX-015 is an oncolytic adenovirus that lacks the E1B-55K gene product required for p53 degradation and therefore was predicted to selectively replicate in tumor cells with inactive p53 pathways [5]. Later studies revealed that p53-independent effects may function as regulators of virus replication supporting the therapeutic application of ONYX-015 not only in p53-defficient tumors, but also in tumors with wild-type p53 [6], [7]. ONYX-015 has been tested extensively; evidence for specific oncolysis was found in several clinical trials and in various tumors types [8]–[11], including recurrent head and neck [12], colorectal [13], ovarian [14], and hepatobiliary [11] cancers. Although clear antitumor activity was demonstrated using ONYX-015 in murine models of cancer, both *in vitro* and *in vivo*, its clinical efficacy in human trials has failed to fulfill the high expectations that were based on animal model studies. A potential explanation for limited activity is reduced expression of the main receptor for adenoviruses, CAR, which is required for efficient virus entry into target cells. Reduced expression of the CAR protein on the cancer cell surface is possibly a result of the epithelial-mesenchymal transition [15].

In previously published work, we explored the possibility of pharmacologically up-regulating CAR in colon cancer cell lines through inhibition of signal transduction pathways involved in its repression. We were able to demonstrate that inhibition of MEK, as well as TGFβ, up-regulates CAR expression *in vitro* and results in enhanced adenovirus entry into the cells [15], [16]. Although disruption of signaling through the RAF-MEK-ERK pathway restores CAR expression, it potentially interferes with the replication of ONYX-015 due to G1-phase cell cycle arrest, since the virus has demonstrated sensitivity to the cell cycle phase of infected cells [17], [18]. Thus, optimization of this combination treatment strategy is difficult since the effects of MEK inhibitors, as well as the interaction of adenoviruses with target cells, are highly complex, dynamic, and non-linear processes. Through mechanistic modeling of cancer cells subject to MEK-inhibition and ONYX-015 infection, we seek to characterize and predict system dynamics in order to improve the efficacy of oncolytic adenovirus cancer treatment by manipulating the timing of MEK-inhibitor treatment and oncolytic adenovirus infection. Through successful test of model predictions, our goal is to elucidate *in vitro* strategies that could offer practical and effective means for minimizing cancer growth *in vivo*. Our studies provide a paradigm for the development of optimized combination therapies for cancer through experimentally validated mathematical modeling of non-intuitive behavior of cancer cells.

## Results

### *In vitro* quantification of CAR expression, cell proliferation, infection, cell viability, and viral replication time courses motivate model development

**Figure 1:**
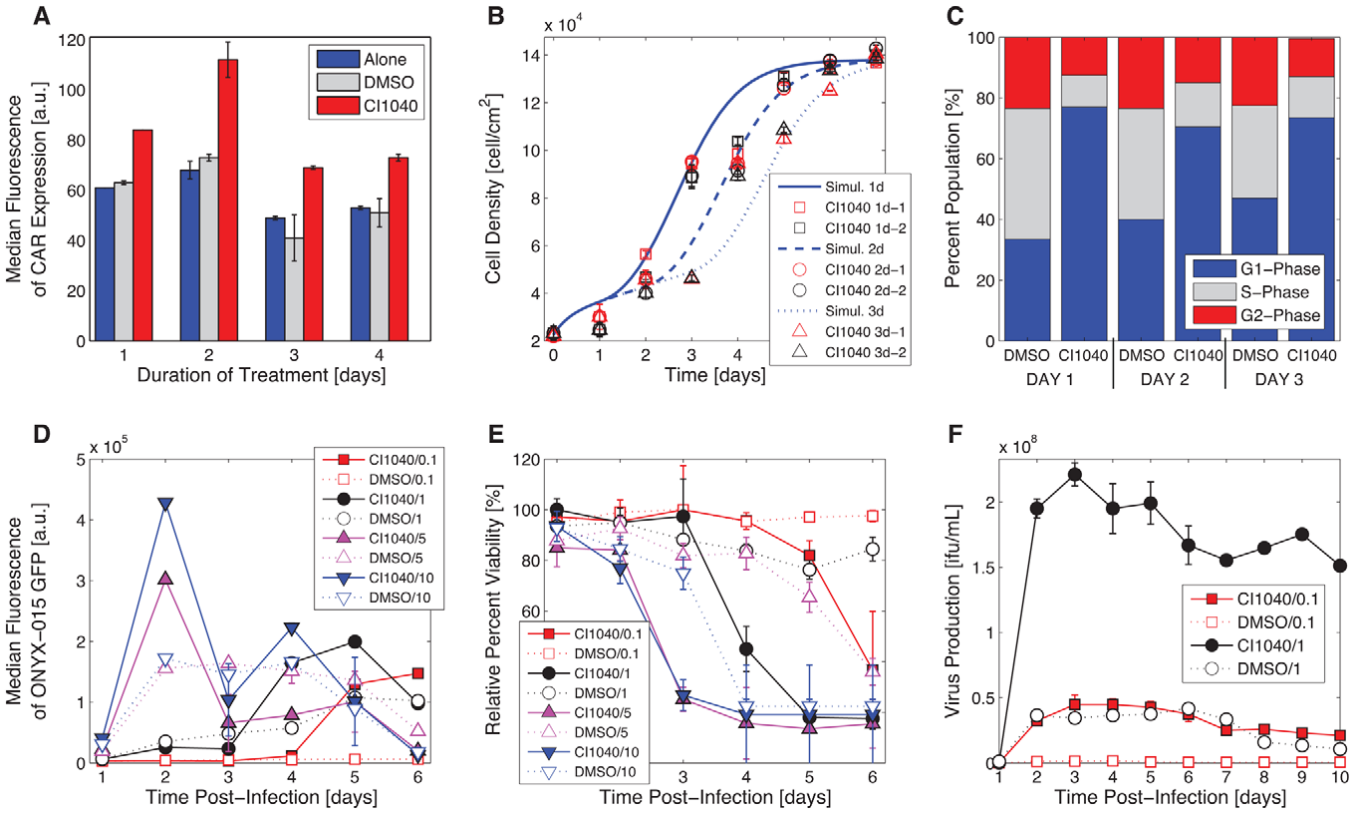
CI1040 up-regulates CAR, induces G1 cell cycle arrest, and sensitizes cells to infection. HCT116 cells were treated with MEK inhibitor CI1040, DMSO, or alone. (**A**) Treatment was continuous for 4 days. CAR expression was measured 1–4 days post-treatment initiation by FACS; error bars represent standard deviation of triplicate measurements. (**B**) Cells were treated with CI1040 for 1 day, 2 days, or 3 days, and harvested 1–7 days following initial treatment. Cell density was determined (red and black data markers); error bars represent standard deviation of triplicate measurements. Each time course was replicated. Solid blue lines correspond to simulated proliferation dynamics with respective CI1040 treatment. (**C**) Cell cycle phase was measured 1–3 days post treatment with CI1040 or DMSO through PI staining. (**D–E**) Cells were treated with CI1040 or DMSO for 2 days, treatment is removed by media change, and cells were immediately infected with ONYX-015/GFP (D) or ONYX-015 (E) at MOIs 0.1, 1, 2 (not shown), 5, and 10. GFP expression (determined by FACS analysis) and cell viability was measured 1–6 days post-infection. (**F**) Cells were treated with CI1040 for 2 days, treatment was removed by media change, and cells were immediately infected with ONYX-015 at MOIs of 0.1 and 1. Virus replication was observed 1–10 days post infection.

In order to generate sufficient experimental data quantifying the mechanistic behavior critical to predicting nonlinear dynamics, we systematically assessed CAR expression, cell proliferation, infection, cell viability, and viral replication in the presence and absence of MEK inhibitors (namely, CI1040). In agreement with our previously published work [16], we found that disrupting the MAPK signaling pathway through pharmacological inhibition of MEK nearly doubles the number of CAR molecules per cell relative to the control (DMSO-treated) cells. The largest increase in receptor levels occurred 2 days after CI1040 treatment initiation (Figure 1a). Such restoration also presented a tradeoff: MEK-inhibition caused G1-phase cell cycle arrest (Figure 1b and 1c). Previous studies indicated that cell cycle arrest inhibits production of new virus particles and virus replication [17]. We therefore hypothesized that effective oncolytic adenovirus infection requires pre-treatment of cells with MEK inhibitor for a sufficient amount of time, providing increased receptor expression at the cell surface. To allow the cell cycle to proceed, treatment should be followed by removal of the inhibitor at the time of infection. Thus, we pre-treated cells with either CI1040 or DMSO for 2 days prior to infecting cells at multiplicities of infection (MOIs) of 0.1, 1, 2, 5, and 10. We observed increased infection (Figure 1d) and found that viability of HCT116 cells pre-treated with CI1040 decreased by 60% or 65% six days post infection following MOIs of 0.1 and 1 (respectively) when compared to the DMSO control (Figure 1e). At higher MOI, pre-treatment with MEK-inhibitor accelerated cell killing by as much as 3 days. In agreement with the observed enhanced cell death upon CI1040 pre-treatment, virus production also improved: virus titer increased 20-fold at MOI of 0.1 and 5-fold at MOI of 1 when measured five days post-infection (Figure 1f). Our findings suggest that treating cells with MEK-inhibitor prior to infection increases CAR expression, arrests cells in G1 cell cycle phase, and sensitizes cells to infection such that we observe reduced viability and improved virus replication.

### A nonlinear differential equation model characterizes the combinatorial effect of MEK-inhibition and oncolytic adenovirus infection on cancer cell populations

We sought to build a model that captures the key phenotypic behavior of tumor cells responding to combinatorial therapy. We fit an ordinary differential equation (ODE) model to measurements of proliferation, infection, and relative cell viability, characterizing how an *in vitro* cancer cell population responds to MEK inhibition and ONYX-015 infection. Quantitative time course data supported development of a 4-state nonlinear ODE system with treatment- and infection-dependent parameter values (Figure 1). In this context, state variables represent observable tumor cell conditions in response to MEK-inhibition and/or adenovirus infection.
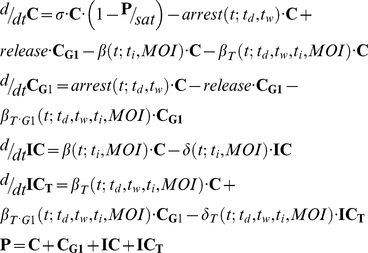



**Figure 2:**
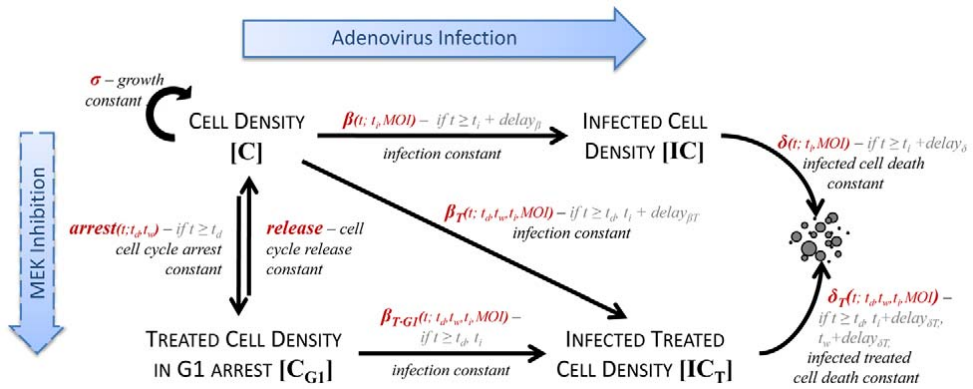
Experimental observations motivate a nonlinear ordinary differential equation model for cancer therapy. System states (shown in black bold capital font) represent the nonlinear dynamic behavior of (i) uninfected cancer cell density, **C** [cells/cm2], (ii) MEK-inhibition induced G1 arrest cell density, **C_G1_** [cells/cm2], (iii) untreated and infected cell density, **IC** [cells/cm2], and (iv) treated and infected cell density, **IC_T_** [cells/cm2]. Parameter values (shown in red italic script) govern treatment/infection dependent state transitions (solid black arrows) that direct proliferation (*σ*), G1 cell cycle arrest/release, infection (*β_n_*), and lysis (*δ_n_*), where *n* denotes whether these cells infect/lyse from a treated (*n* = T or *n* = T·G1) or untreated (*n* = ‘blank’) state. Corresponding delay terms are shown in gray font. MEK-inhibition is described as a reversible process since cells undergo G1 arrest via CI1040 treatment and release upon removal of MEK-inhibitor by media change, returning to the proliferating state (dashed block arrow). Infection is an irreversible process that ultimately results in cell death (solid block arrow).

System states are depicted in black bold capital font (Figure 2) and reflect the nonlinear dynamic behavior of (i) uninfected cancer cell density, **C** [cells/cm^2^], (ii) MEK-inhibition induced G1-phase arrest cell density, **C_G1_** [cells/cm^2^], (iii) untreated and infected cell density, **IC** [cells/cm^2^], and (iv) MEK-inhibitor treated and infected cell density, **IC_T_** [cells/cm^2^], where **P** reflects the total cancer cell population [cells/cm^2^].

Corresponding parameter values govern the rate at which state variables proliferate, arrest in (release from) the G1 cell cycle phase as a result of MEK-inhibitor treatment (removal), infect, and lyse (Figure 2). Specifically, parameter *σ* governs the rate at which cells proliferate until they reach 100% confluence at the threshold, *sat* [cells/cm^2^]. Parameters *β_n_* govern the rate at which cells irreversibly undergo ONYX-015 infection, where *n* denotes whether these cells infect from a treated (*n* = T or *n* = T·G1) or untreated (*n* = ‘blank’) state. Since pre-treatment with MEK-inhibitor enhanced infection and cell killing, we presumed that cells in the arrested **C_G1_** state were more susceptible to infection due to increased CAR expression, motivating the transition directed by parameter *β_T·G1_* (Figure 2). Experimental data suggests, however, that a fraction of cells responding to MEK-inhibitor do not undergo cell cycle arrest (Figures 1b and 1c). Despite continued proliferation, this subpopulation might exhibit increased CAR expression. For this reason, we incorporated parameter *β_T_*, allowing a subpopulation of cells (assumed to have increased CAR expression) to transition from the proliferating state, **C**, to the infected treated state, **IC_T_**, upon MEK-inhibition and ONYX-015 infection. Parameters *δ_n_* govern the rate at which cells undergo oncolysis.

#### Parameter estimation and model structure

Parameter estimation involves changing the model's parameter values until the difference between the model output (i.e., simulation) and experimental data is minimized as defined by the sum of squares error, SSE, weighted by the measurement error associated with each data point. The weight corresponds to the inverse standard deviation of replicate measurements. If the standard deviation is relatively small, our confidence in the measurement is high, so we penalize the simulated error with greater magnitude. Weighted SSE was employed when fitting proliferations kinetics since multiple data replicates were available; standard SSE was employed when fitting infection and viability kinetics (Text S1). Fitting system parameters is nontrivial since their values can change with respect to the timing of drug treatment and infection; parameters are not constant in time. In most cases, they are defined as functions of the system's inherent control inputs: timing of MEK-inhibitor treatment initiation, *t_d_*; timing of MEK-inhibitor removal by media change, *t_w_*; timing of infection, *t_i_*; and multiplicity of infection, *MOI*. In addition to fitting dynamic parameters, we estimated the value of time delays associated with infection and oncolysis.

We expect delays throughout the infection cycle: endocytosis, viral replication, and lysis require a sequence of non-instantaneous sub-cellular events [4] that are typically omitted from cell-level models. We accounted for the dynamics of these events by introducing time delays. Specifically, we fit parameters to several forms of the model and evaluated the accuracy of simulations. While each system included parameters governing proliferation, infection, and cell death, they differed on whether they contained explicit time delays. For instance, some models stipulated that *β_n_* and/or *δ_n_* remain 0 until *t*≥*t_i_*+*delay*. After fitting parameters to each possible delay combination, we retained the model structure that best fit the data (Text S1).

#### Simulated dynamics

Model parameters were estimated in sequence to improve the biological relevance of fitted values while emulating experimental conditions. Most resulting parameters were defined as functions of the system's inherent control inputs, where the duration of treatment is set to 2-days: *t_w_* = *t_d_*+2. Proliferation-related values (namely, *σ* and *sat* for the DMSO case, and *arrest* and *release* for the CI1040 case) were fit first to a reduced ODE system. Once each parameter was optimized, we quantified the precision of resulting simulations by evaluating the average normalized error of the time course. Resulting simulations were, on average, within 8% (DMSO) and 6% (CI1040; Figure 1b) of the mean of experimental data replicates. Upon setting *σ*, *sat*, *arrest*, and *release*, a second reduced model (characterizing **C** and **IC**) was used to fit *β*, *δ*, and corresponding *delay* terms to experimental data quantifying virus uptake and cell viability kinetics with DMSO treatment. Parameter estimation yielded four unique values per MOI with corresponding simulations within 9% of experimental measurements. Finally, with proliferation and DMSO-related parameters fixed, the full 4-state model was used to fit *β_T_*, *β_T·G1_*, *δ_T_*, and corresponding *delay* terms to experimental data quantifying virus uptake and cell viability kinetics with CI1040 treatment. Parameter estimation yielded five unique values per MOI with corresponding simulations within 8% of experimental measurements. Please refer to Text S1 for details concerning parameter estimation and resulting values.

#### Model assumptions

We made several assumptions to simplify model development and supporting experiments. First, we neglected state transitions between cell cycle phases and characterized only the switch between proliferating cells and cells arrested in G1-phase as a result of treatment with MEK-inhibitor. Regarding enhanced infection and cell death, we presumed that MEK-inhibition caused a switch-like sensitivity to infection instead of a linear progression. Therefore, if 100% of cells were arrested in G1-phase as a result of MEK-inhibition, prolonged treatment would not further enhance infection. We neglected dose and duration of treatment as system control variables and limited simulated MOI between 0.1 and 10 to avoid error due to extrapolation. We assumed cancer cell populations were spatially uniform such that experimental measurements reflected a deterministic (rather than spatial or stochastic) mean behavior. Finally, we did not explicitly characterize virus titer. Instead, the presumed effects of virus dynamics were consolidated into estimated parameters.

### Validation of simulated predictions reveals optimized treatment sequencing protocols

**Figure 3:**
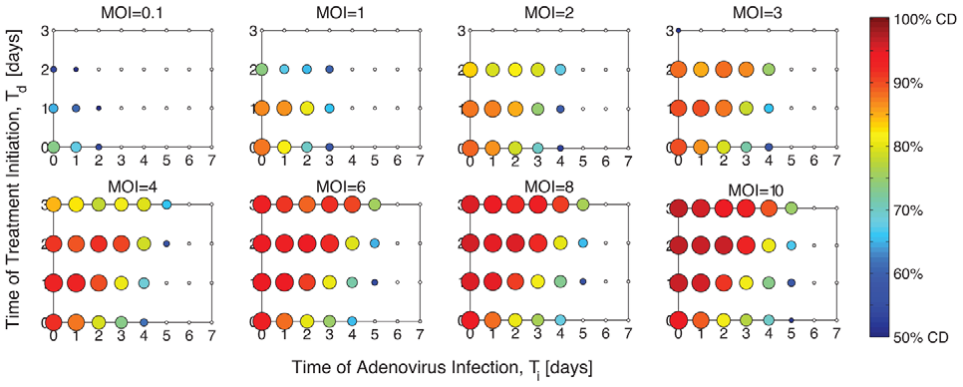
Simulated predictions point to unexpected treatment protocols. Simulated percent cell death (CD) is evaluated on day 8 as a function of the timing of MEK-inhibitor treatment initiation, timing of infection, and multiplicity of infection (MOI). Each Cartesian coordinate reflects an independent simulation or treatment/infection protocol. The timing of ONYX-015 infection is varied on the x-axis; the timing of CI1040 treatment initiation is varied on the y-axis. CI1040 removal by media change occurs 2 days post treatment irrespective of the timing of infection. MOI is held constant in each subplot. Percent cell death is defined as the complement of cell viability. Treatment and infection protocols that yield over 50% cell death are shown. Greater cell death is reflected by larger data points and an increasingly red color (see color bar). Empty data points depict protocols that fail to kill at least 50% of the cellular population.

We interpolated intermediate parameter values and used the model to predict the extent of cell death as a function of the time of CI1040 treatment initiation, the time of ONYX-015 infection, and the MOI. We employed an exhaustive search algorithm to simulate the effect of various treatment and infection protocols. This algorithm systematically evaluated every possible sequence combination of drug treatment and infection conditions (within a defined interval), with the exception of media change, *t_w_*, which was set to occur 2 days after treatment. We varied CI1040 treatment initiation between days 0–3 and infection between days 0–7. The MOI was also varied between 0.1 and 10. We evaluated percent cell death on day 8 irrespective of the sequence protocol. In this context, percent cell death is defined as the complement of cell viability (the ratio of total cell density in a simulation consisting of treatment and infection, relative to total cell density in an independent simulation omitting infection). In Figure 3, we highlight drug treatment and infection protocols that yielded over 50% cell death on simulated day 8. (Please refer to Text S1 for additional MOI). Model simulations suggested that, at low MOI, the greatest efficacy of virus-mediated cell death results from MEK-inhibition that coincides with the time of infection. At higher MOI, our model predicted maximal cell killing when inhibitor treatment occurs at the time of, or soon after, infection.

**Figure 4:**
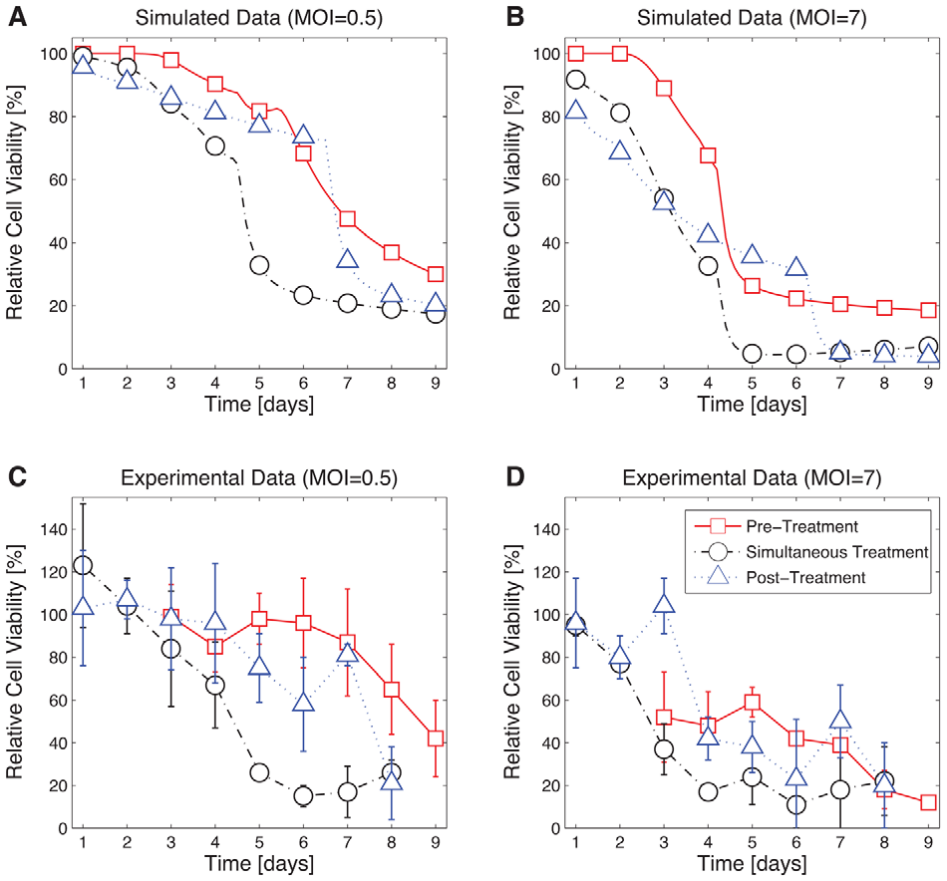
Simultaneous CI1040 treatment and infection protocols outperform pre-treatment with CI1040. HCT116 cells were treated with CI1040 or DMSO, and infected with ONYX-015 at MOI = 0.5 or MOI = 7. The cell viability outcome of three different treatment protocols is compared in each plot: *pre-treatment*, *simultaneous treatment*, and *post-treatment*. In the first case (depicted by square markers), cells are treated on day 0, treatment is removed by media change on day 2, and immediately infected. In the second case (circle markers), cells are treated and infected simultaneously on day 0, and treatment is removed by media change on day 2. In the third case (triangle markers), cells are infected on day 0, treated on day 2, and treatment is removed by media change on day 4. Model simulations predicting the response of cells to infection at MOIs 0.5 and 7 are shown in (**A**) and (**B**), respectively. Experimental validation of predicted cell viability for (**C**) MOI = 0.5 and (**D**) MOI = 7 was measured daily 7 days post infection (for pre-treatment protocols) and 8 days post-infection (for simultaneous and post-treatment protocols). The figure legend is consistent among all plots.

To experimentally validate the predictive capabilities of the model, we simulated (Figures 4a and 4b) and experimentally quantified (Figures 4c and 4d) cell viability for three distinct drug treatment and infection protocols that employed MOIs not included in the original training data: MOIs of 0.5 and 7. Specifically, we compared (i) *pre-treatment* with the MEK inhibitor on day 0 followed by media change and immediate infection on day 2, (ii) *simultaneous* drug treatment and infection on day 0 followed by media change on day 2, and (iii) infection on day 0 followed by *post-treatment* initiation on day 2 and media change on day 4. Cell viability was quantified daily post-infection until day 7. The mean error between simulations and time course measurements were promising: *pre-treatment* simulations were within 19% of validation data for both MOIs; *simultaneous* treatment simulations were within 8% and 12% of validation data for MOIs of 0.5 and 7, respectively; and *post-treatment* simulations were within 14% and 19% of validation data for MOIs of 0.5 and 7, respectively. One cause of disparity between predictions and experimental data relates to modeling constraints. The ODEs are formulated such that resulting simulations cannot yield more than 100% viability post-infection. Some experimental measurements, however, reflected an initial increase in cell viability that was observed in replicate measurements. This increase may result from viral proteins that activate cellular factors and force cells to enter S-phase and replicate. Despite this lack of correspondence, experiments and simulations share similar qualitative and quantitative time course dynamics, confirming that our model is indeed predictive, and that simultaneous treatment and infection significantly improves oncolysis.

### Efficacy of MEK-inhibition mediated infection is inversely correlated with cell confluency at the time of infection

**Figure 5:**
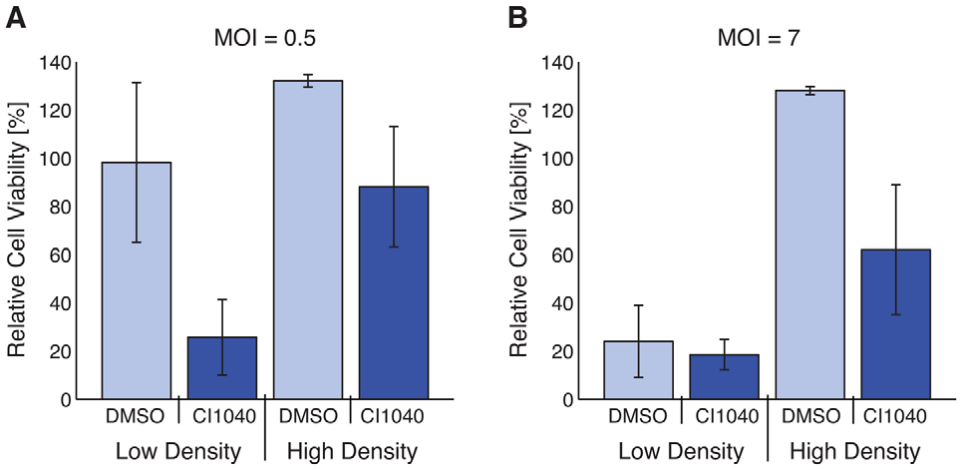
Efficacy of treatment/infection correlates to cell density at the time of infection. HCT116 cells were seeded at 2e4 cells/well (low density) or 1e5 cells/well (high density) in 96-well plates. Low and high density cells were treated with DMSO or CI1040, and infected at an MOI of (**A**) 0.5 or (**B**) 7. Cell viability was quantified 3 days post infection.

To further investigate conditions that give rise to increased therapeutic efficacy, we correlated simulated cell death profiles with (i) cell confluency at the time of treatment, (ii) the proportion of cells in CI1040 mediated G1 cell cycle arrest at the time of infection, and (iii) cell confluency at the time of infection. Little correlation between cell death and cell confluency at the time of drug treatment was found by Pearson correlation analysis (R = .2; *p*-value≪0.001). Additionally, little correlation was found between cell death and the proportion of cells in G1-phase arrest at the time of infection, C_G1_/P (R = .3; *p*-value≪0.001). Despite these weak correlation coefficients, simulations suggest that cell killing is inversely correlated with cell density at the time of infection (R = 0.6; *p*-value≪0.001). Thus, greater cell density at the time of infection may decrease the efficacy of infection despite consistent treatment/infection strategies. This result is reasonable since standard protocols suggest that the confluency for infection be approximately 70–80% [19]. We experimentally validated this finding, *in vitro*, by measuring cell viability after infection of cells that were seeded at low (∼70%) and high (∼100%) confluencies (Figure 5). We found that a lower cell density at the time of infection resulted in greater cell killing, supporting the notion that cell confluency might regulate the efficacy of infection and oncolysis.

### MEK-inhibition offers CAR-independent amplification of oncolysis and virus production *in vitro*

**Figure 6:**
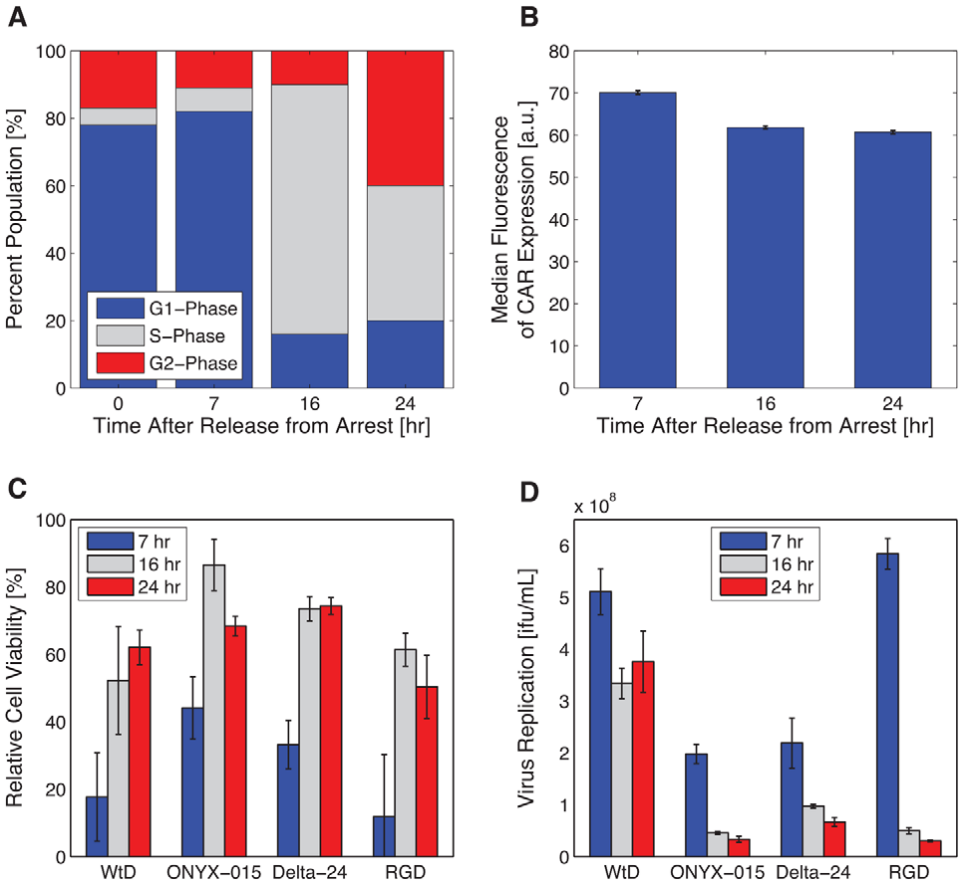
G1 arrested cells show more potent cell killing effect and virus production. HCT116 cells were density arrested and released from synchronization. (**A**) Cell cycle distribution was quantified upon release at 0, 7, 16, and 24-hours. (**B**) The effect of cell cycle synchronization on CAR expression was analyzed. (**C**) Cell viability and (**D**) virus production were measured 3 days after infection (MOI = 1) with WtD, ONYX-014, Delta-24, and Delta-24RGD (denoted as RGD) at 7, 16, and 24-hours after release from density arrest. Error bars represent the standard deviation of triplicate measurements for cell viability and duplicate measurements for virus production.

Model simulations and experimental validation confirm that simultaneous treatment with MEK-inhibitor and infection is most advantageous, suggesting that alternate (CAR-independent) regulatory mechanisms may be responsible for enhanced oncolysis. Given the disproportionate increase in virus replication relative to infection (Figure 1e), we hypothesized that enhanced CAR may not be the key factor involved in amplifying virus replication and cell death; MEK-inhibition might provide an alternate mechanism responsible for greater efficacy of infection. Since MEK inhibitor treatment leads to G1-phase cell cycle arrest in HCT116 cells, we tested the impact of cell cycle distribution on oncolysis and virus production in cells infected with various oncolytic adenoviruses. HCT116 cells were arrested in the G1-phase of the cell cycle by contact inhibition and released by re-seeding at sub-confluent densities. We then quantified the change in cell cycle distribution as cells transitioned from the G1-phase. At 7-hours after re-seeding, 80% of the cell population remained in G1-phase. At 16-hours, 80% of cells reached S-phase (Figure 6a). Meanwhile, CAR expression remained unchanged throughout these cell cycle phase transitions (Figure 6b). Despite constant CAR, we observed significant differences in cell killing when infection occurred at 7-hours, 16-hours, and 24-hours after re-seeding. The greatest lytic effect occurred when infection took place 7-hours after re-seeding (Figure 6c), which coincided with greatest virus production (Figure 6d). The marked increase in cell death and adenovirus replication suggests that the G1-S phase transition mediates adenovirus replication. These observations were confirmed with a variety of oncolytic adenoviruses, including Delta-24RGD, which is characterized by an RGD motif on the fiber knob of the adenovirus allowing for CAR-independent infection [20]. Therefore, the expression of CAR molecules on the cell surface at the time of infection does not appear to be the sole regulatory mechanism governing the efficacy of oncolytic adenovirus therapy. The detailed molecular mechanisms by which cell cycle distribution influences viral replication are currently under investigation.

## Discussion

A growing number of studies make use of mathematical modeling techniques to better analyze and predict increasingly complex, dynamic data. While several groups have employed computational approaches to optimize oncolytic virotherapy [21]–[25], only two other groups have investigated the combinatorial dynamics that govern MEK-inhibitor mediated oncolytic adenovirus therapy [26], [27]. Here, we report our findings on improved treatment strategies for oncolytic adenovirus therapy, being the first to fully integrate modeling and experiment in the same study.

We performed time course measurements that confirmed previously observed CI1040-mediated CAR up-regulation and G1 cell cycle arrest [28]. Based on these findings, we postulated that treating cells with CI1040 prior to infection, followed by its removal at the time of ONYX-015 infection, would (i) maximize virus uptake due to increased up-regulation of CAR, and (ii) maximize cell death (and consequently viral replication) due to the release of cells from G1-phase arrest. To explore this hypothesis, we developed an ODE model that characterized the proliferation, infection, and relative cell viability of a population of cancer cells subjected to MEK inhibition and ONYX-015 infection. We simulated combinations of different timings of MEK-inhibitor treatment initiation, timings of infection, and multiplicities of infection to ascertain their combinatorial effect on oncolysis. Surprisingly, our simulations suggested that, at low MOI, the greatest efficacy of virus-mediated cell death results from MEK-inhibition that coincides with the time of infection. This scenario is particularly relevant from a clinical perspective, since exposure to low MOIs is a likely limiting factor of treatment efficacy *in vivo*, particularly following systemic virus administration. At higher MOI, our model predicts maximal cell killing when inhibitor treatment occurs at the time of, or immediately after, infection. We confirmed our predictions experimentally, showing that sensitizing cells via MEK-inhibition *prior* to infection was less effective than treatment protocols that maintained CI1040 treatment during and following ONYX-015 infection. Consistent with our findings, simulations from an independent partial differential equation free boundary problem model presented by Tao and Guo [26] suggested that greater tumor treatment is achieved when oncolytic adenovirus infection and MEK inhibitor treatment occur at the same time. Experimental validation was not carried out in that study.

The accuracy between simulated time courses and validation measurements (Figure 4) were striking given that validation conditions were beyond the scope of training data. More specifically, we predicted cell dynamics associated with MOIs 0.5 and 7; neither condition was considered in model development. Data reflecting MOIs 0.1, 1, 2, 5, and 10 were used for parameter estimation and subsequently interpolated to predict intermediate values. Furthermore, model fitting was based on time course measurements quantifying the effect of pre-treatment with MEK inhibitors (or DMSO) followed by infection, yet we are able to extrapolate cell viability dynamics for mechanistically unique protocols. Specifically, the *simultaneous* and *post-treatment* protocols involve experimental procedures that were unaccounted for in model development. Simulations also assumed that the MOI of the system remained unaffected upon removal of MEK-inhibitor by media change. This theory is accurate when treatment occurs prior to infection (as was the case in our training data). When treatment occurs at the time of or after infection, it is reasonable to imagine removal of MEK-inhibitor affecting virus titer, and hence the MOI. Despite these differences, model simulations accurately predict and extrapolate the nonlinear cellular response to combinatorial treatment strategies.

Further investigations of simulated predictions identified critical virus-host mechanisms responsible for enhanced combinatorial therapy. In particular, we explored how cell cycle phase affected oncolysis and virus production. Shepard and Ornelles [29] demonstrated that ONYX-015 replicates more effectively in HeLa cells when infection occurs during S-phase rather than G1-phase. Later, Zheng *et al.* [30] found that adenovirus E1B55K is required to enhance cyclin E expression; the failure to induce cyclin E expression due to E1B55K mutation in ONYX-015 prevents viral DNA from undergoing efficient replication in HeLa (and other non-permissive) cells when infected during G0-phase. In contrast, cyclin E induction is less dependent on the function encoded in the E1B55K of HCT116 cells whether the cells are in S- or G0-phase. This finding is consistent in other cancer cells that are permissive for replication of ONYX-015. As a result, we expanded our analysis to two additional oncolytic adenoviruses: Delta-24, which carries a deletion in the E1A region; and Delta-24RGD, which has an RGD-4C peptide motif inserted into the adenoviral fiber [20]. The latter virus is able to anchor directly to integrins, providing CAR-independent mechanisms for infection. We found greater cell killing and virus production with Delta-24RGD when cells were infected during G1 cell cycle arrest. This result verifies the existence of a regulatory pathway that governs virus production in a CAR-independent manner. The mechanism underlying virus replication in G1-arrested cells remains unclear and warrants further investigation.

Model development is an ongoing process that needs to be tightly coupled with experiments in order to maximize mechanistic relevance and reflect the nonlinear complex dynamics critical to understanding and predicting biological function. However, it is important to note that our current model does not fully encompass the physiological complexities of malignant tumors in humans. It is clear that factors influencing drug distribution and elimination play a major role in this context. For example, the extent of vascular leakiness observed in tumors will impact viral extravasation [31]. The immune responses directed against oncolytic viruses or tumor cells will also impact viral anti-tumor effects [25]. Interestingly, recent *in vivo* experiments demonstrate that the efficacy and specificity of virus replication in tumors modulate the immune response, highlighting yet another layer of complexity [32]. Our aim is to develop multi-scale models that account for the greater dimensionality of oncolytic virus replication. Nevertheless, our current study demonstrates that dynamical mathematical models of oncolytic virus replication, tightly coupled with experimental studies, have the potential to optimize central aspects of this therapeutic approach.

## Materials and Methods

### Cell lines

The colon cancer cell line, HCT116, was kindly provided by Dr. B. Vogelstein (Johns Hopkins Cancer Center, Baltimore, MD). HCT116 cells were cultured in McCoy's 5A medium (UCSF Cell Culture Facility, San Francisco, CA) supplemented with 10% fetal bovine serum (Valley Biomedical Products, Winchester, VA).

### Adenoviruses

Viruses included a wild-type adenovirus, WtD; an E1B-55K-deficient adenovirus mutant, ONYX-015; an E1A-deficient adenovirus, Delta-24 [33]; and a modified version of Delta-24 containing an RGD-4C peptide motif inserted into the adenoviral fiber knob which allows the adenovirus to anchor directly to integrins, Delta-24RGD [20]. Delta-24 and Delta-24RGD were kindly provided by Dr. J. Fueyo (University of Texas MD Anderson Cancer Center, Houston, Texas). Adenoviruses were amplified in HEK-293 cells, purified using the Adenopure Purification Kit (Puresyn, Malvern, PA) and their titers determined using the Adeno-X Rapid Titer Kit (Clontech, Mountain View, CA). ONYX/GFP, a green fluorescence protein expressing ONYX-015 was also used.

### Signal transduction inhibitors

For inhibition of RAF-MEK-ERK signaling, the MEK inhibitor CI1040 (Pfizer, Ann Arbor, MI) was used at a final concentration of 5 µM. As a control, cells were treated with DMSO (0.1%).

### CAR and GFP expression by flow cytometric analysis

For CAR staining, cells were treated with CI1040, DMSO, or cell culture medium alone (as stated previously). Over the course of 4 days, the cells were harvested daily using 0.05% trypsin (UCSF, Cell Culture Facility, San Francisco, CA). After media change in PBS (UCSF), cells were incubated for 45 minutes at 4°C with the mouse monoclonal anti-CAR antibody RmcB (1∶50) [34]. After washing, cells were incubated for 30 minutes at 4°C with a secondary antibody conjugated to Alexa 488 (1∶100, Alexa Fluor 488 F(ab)2 fragment of goat anti-mouse IgG, Invitrogen, Molecular Probes, Eugene, OR). Propidium iodide (PI, Sigma-Aldrich Co, St Louis, MO) was added to a final concentration of 1 µg/mL just prior to acquisition to exclude dead cells from flow cytometric analysis. Stained cells were analyzed on a FACSCalibur cytometer (Becton Dickinson, Franklin Lakes, NJ). To monitor virus replication in living cells, HCT116 cells were treated with CI1040 or DMSO for 2 days and later infected with ONYX-015/GFP at multiplicities of infection (MOI) of 0.1, 1, 2, 5 and 10 in infection medium; McCoy's5A medium (UCSF) was supplemented with 2% fetal bovine serum (Valley Biomedical Products, Winchester, VA). The medium was replaced two hours later. Cells were harvested 1 to 6 days post-infection using 0.05% trypsin (UCSF) and washed once with PBS (UCSF) supplemented with 5% fetal bovine serum (Valley Biomedical Products). GFP expression was analyzed by a C6 Flow Cytometer (Accuri Cytometer). Text S1 describes methodological details for the flow cytometry experiments, including controls, in accordance with the Minimum Information About a Flow Cytometry Experiment (MIFlowCyt) protocol established by Lee et al. [35].

### Cell cycle and proliferation

For cell proliferation, HCT116 cells were seeded in 6-well plates and immediately treated with CI1040 or DMSO (as stated previously) for 1, 2, or 3 days and harvested 1–7 days after treatment. Cells were counted using a C6 Flow Cytometer (Accuri Cytometer). Treated cells were also analyzed for cell cycle distribution (please refer to Text S1 for details concerning flow cytometry experiments). After treatment, the cells were collected by trypsinization, fixed in 70% ethanol, washed in PBSTB (PBS+0.5% Tween 20 (National Diagnostics, Atlanta, GA) +0.1% BSA (Sigma)), and re-suspended in 350µl of PBSTB containing 0.6 mg/mL RNase and 30 µg/mL PI. Cells were incubated in the dark for 30 min at room temperature and then analyzed by a FACSCalibur cytometer (Becton Dickinson). The data were analyzed using ModFit LT (Verity Software House). Please refer to Text S1 for additional details.

### Cell viability

HCT116 cells were seeded in 96-well plates overnight and infected with WtD, ONYX-015, Delta-24, or Delta-24RGD at different MOI. Cell viability was measured by the CellTiter 96 Aqueous One Solution Cell Proliferation Assay (MTS) (Promega, Madison, WI) 1 to 7 days post-infection. Cell viability was expressed as percentage of the uninfected medium control (i.e. MOI = 0). Therefore, any relevant toxic effects are normalized from the relative viability measurements.

### Cell-cycle synchronization

HCT116 cells were density-arrested by plating at 5e5 cells/cm^2^ for 2 days. Cells were released from arrest by re-plating at low density, 1e5 cells/cm^2^. Cell cycle was analyzed by Propidium Iodide (Sigma) staining as described above. CAR expression was analyzed at 7, 16, and 24 hours after release from arrest using RmcB antibody as described above. Synchronized cells were infected with WtD, ONYX-015, Delta-24, or Delta-24RGD at an MOI of 1 and subsequently measured for viability by adding Propidium iodide (Sigma) to a final concentration of 1 µg/mL just prior to acquisition to exclude dead cells and counting cell numbers using a C6 Flow Cytometer (Accuri Cytometer). Cell viability was expressed as percentage of the uninfected medium control (i.e. MOI = 0).

### Viral replication assays

Viral titers of harvested cells were determined by the Adeno-X Rapid Titer Kit (Clontech) as described by Shiina et al. [36].

### Parameter estimation

Parameters were fit to experimental measurements by minimizing the sum of squares error (SSE) between the simulation and the data using the genetic algorithm function in MATLAB. When multiple data replicates were available, the SSE was weighted by the inverse standard deviation of experimental measurements. A gradient search algorithm, fmincon, was used post-estimation to ensure convergence to a local minimum. Please refer to Text S1 for details concerning parameter estimation, convergence, model fitness, and interpolation methods.

## Supporting Information

Text S1 Contents: 1) parameter estimation; 2) parameter convergence; 3) model fitness; 4) interpolation methods; 5) model simulations; 6) MATLAB syntax for ordinary differential equation model; 7) MIFlowCyt outline.
(0.89 MB PDF)
