# Supplementary material for: A Dynamical Systems Model for Combinatorial Cancer Therapy Enhances Oncolytic Adenovirus Efficacy by MEK-Inhibition
Source: PLoS Comput Biol. 2011 Feb 17;7(2):e1001085. doi: 10.1371/journal.pcbi.1001085 (PMC3040662; doi:10.1371/journal.pcbi.1001085)
Supplement: Text S1 — Contents: 1) parameter estimation; 2) parameter convergence; 3) model fitness; 4) interpolation methods; 5) model simulations; 6) MATLAB syntax for ordinary differential equation model; 7) MIFlowCyt outline. (0.89 MB PDF) [file pcbi.1001085.s001.pdf]

Supplemental Text

A Dynamical Systems Model for Combinatorial Cancer Therapy Enhances Oncolytic Adenovirus Efficacy by MEK-inhibition

Neda Bagheri[1], Marisa Shiina[2], Douglas A. Lauffenburger[1], W. Michael Korn[2]
[1]Department of Biological Engineering, Massachusetts Institute of Technology, Cambridge, MA 02139.
[2]Division of Gastroenterology and Helen Diller Family Comprehensive Cancer Center, University of California San Francisco, San Francisco, CA 94115.

Contents

Parameter Estimation 1
Parameter Convergence 5
Model Fitness 6
Interpolation Methods 7
Model Simulations 8
MATLAB Syntax for Ordinary Differential Equation Model 9
MIFlowCyt Outline 11

We fit parameters to experimental measurements by minimizing the weighted sum of squares error (WSSE) or sum of squares error (SSE) between the model output (i.e., simulation) and data using the genetic algorithm function in MATLAB, followed by a local gradient descent search, fmincon. When multiple data replicates were available, the SSE was weighted by the inverse standard deviation of experimental measurements. Thus, WSSE was employed when fitting proliferations kinetics and SSE was employed when fitting infection and viability kinetics. To ensure that resulting parameter rate constants related to biological observations, we fit parameters sequentially by emulating specific experimental protocols. Following multiple iterations of parameter estimation, we analyzed the resulting values in light of the model structure to confirm that our system exhibited no unusual parameter identifiability issues. Prior to our analysis of system dynamics, we assessed model sensitivity with respect to various interpolation methods.

Parameter Estimation

First, we fit nominal proliferation kinetics (governed by parameters in red font) such that corresponding simulations were within 8% of DMSO data (Figure S1): $d\mathbf{C}/dt = \sigma \cdot \mathbf{C} \cdot (1-\mathbf{C}/sat)$. This simplification is warranted since treatment, infection, and virus-induced cell death are excluded from the supporting experiments and corresponding ODEs. Cell growth was limited by a 100% confluence threshold, *sat*, which was also fit to the data.

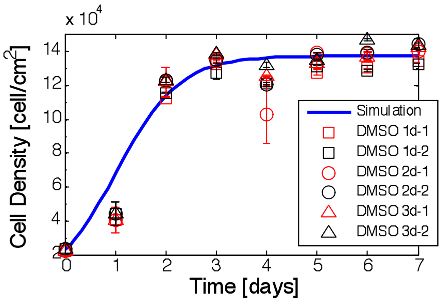

Figure S1: Proliferation kinetics. Cells were treated with DMSO for 1 day, 2 days, or 3 days, and harvested 1-7 days following initial treatment. Cell density was determined (red and black data markers); error bars represent standard deviation of triplicate measurements. Each time course was replicated. Solid blue lines correspond to simulated proliferation dynamics with respective CI1040 treatment.

We fixed the estimated values (Table S1) and fit MEK-inhibition induced G1 cell cycle phase arrest and release parameters (in red) to CI1040 data (Figure 1b). Resulting simulations were within 6% of the corresponding data:

$d\mathbf{C}/dt = \sigma \cdot \mathbf{C} \cdot (1-\mathbf{P}/sat) - arrest(t;t_d,t_w) \cdot \mathbf{C} + release \cdot \mathbf{C_{G1}}$

$d\mathbf{C_{G1}}/dt = arrest(t;t_d,t_w) \cdot \mathbf{C} - release \cdot \mathbf{C_{G1}}$

$\mathbf{P} = \mathbf{C} + \mathbf{C_{G1}}$.

The parameter directing the rate at which cells release from G1 cell cycle arrest, *release*, was evaluated as both a constant parameter and a dynamic function. We retained the constant parameter form of *release* since resulting simulations offered greater precision (i.e., a lower SSE).

Upon fitting σ, *sat*, *arrest*, and *release*, we quantified virus uptake and cell viability using replication-competent ONYX-015 to characterize the infection and cell death dynamics without use of MEK-inhibition (Figure S2). As a result, we were able to use another reduced form of the model to fit β, δ, and corresponding *delay* terms (in red) to DMSO infection data:

$d\mathbf{C}/dt = \sigma \cdot \mathbf{C} \cdot (1-\mathbf{P}/sat) - \beta(t;t_i,\mathrm{MOI}) \cdot \mathbf{C}$

$d\mathbf{IC}/dt = \beta(t;t_i,\mathrm{MOI}) \cdot \mathbf{C} - \delta(t;t_i,\mathrm{MOI}) \cdot \mathbf{IC}$

$\mathbf{P} = \mathbf{C} + \mathbf{IC}$

We fit parameters to different versions of the model maintaining the form that included a unique delay term associated with infection, and a unique delay term associated with cell death. Parameter fitting yielded 4 unique values (per MOI) with corresponding kinetics within 3, 5, 6, 7, and 9-percent of the experimental data for MOIs of 0.1, 1, 2, 5, and 10, respectively (Figure S2; Table S1).

We fixed DMSO specific infection/viability parameters and fit the remaining virus uptake and cell viability parameters to experimental data that quantified the cellular response to replication competent ONYX-015 infection after 2 days of MEK-inhibitor pre-treatment (Figure 1e). This data supported fitting the β_T, $\beta_{T\cdot G1}$, δ_T, and corresponding *delay* terms (in red) associated with the CI1040 portion of the model:

$d\mathbf{C}/dt = \sigma \cdot \mathbf{C} \cdot (1-\mathbf{P}/sat) - arrest(t;t_d,t_w) \cdot \mathbf{C} + release \cdot \mathbf{C_{G1}} - \beta(t;t_i,\mathrm{MOI}) \cdot \mathbf{C} - \beta_T(t;t_d,t_w,t_i,\mathrm{MOI}) \cdot \mathbf{C}$

$d\mathbf{C_{G1}}/dt = arrest(t;t_d,t_w) \cdot \mathbf{C} - release \cdot \mathbf{C_{G1}} - \beta_{T\cdot G1}(t;t_d,t_w,t_i,\mathrm{MOI}) \cdot \mathbf{C_{G1}}$

$d\mathbf{IC}/dt = \beta(t;t_i,\mathrm{MOI}) \cdot \mathbf{C} - \delta(t;t_i,\mathrm{MOI}) \cdot \mathbf{IC}$

$d\mathbf{IC_T}/dt = \beta_T(t;t_d,t_w,t_i,\mathrm{MOI}) \cdot \mathbf{C} + \beta_{T\cdot G1}(t;t_d,t_w,t_i,\mathrm{MOI}) \cdot \mathbf{C_{G1}} - \delta_T(t;t_d,t_w,t_i,MOI) \cdot \mathbf{IC_T}$

$\mathbf{P} = \mathbf{C} + \mathbf{C_{G1}} + \mathbf{IC} + \mathbf{IC_T}$

We fit delay parameters to different versions of the model:

i. Unique delay term for δ_T.
ii. Unique delay terms for δ_T, β_T, and $\beta_{T\cdot G1}$.
iii. Unique delay term for δ_T, and a common delay term for β_T and $\beta_{T\cdot G1}$.
iv. Unique delay terms for δ_T, and $\beta_{T\cdot G1}$.
v. Unique delay terms for δ_T, and β_T.

The model reflecting greatest precision with the data incorporated delays in infection and lysis when the population of cells was not in G1-arrest (v). In other words, model development suggested that cells in CI1040-mediated G1 arrest immediately infect and transition to the treated-infected state, IC_T. Parameter fitting for this case yielded 5 unique values (per MOI) that were accurate to within 5, 8, 8, 7, and 6-percent of the experimental data for MOIs of 0.1, 1, 2, 5, and 10, respectively (Figure S3). The resulting parameters (Table S1) were specific to cells that had been pre-treated with CI1040 for 2 days, followed by media change and infection.

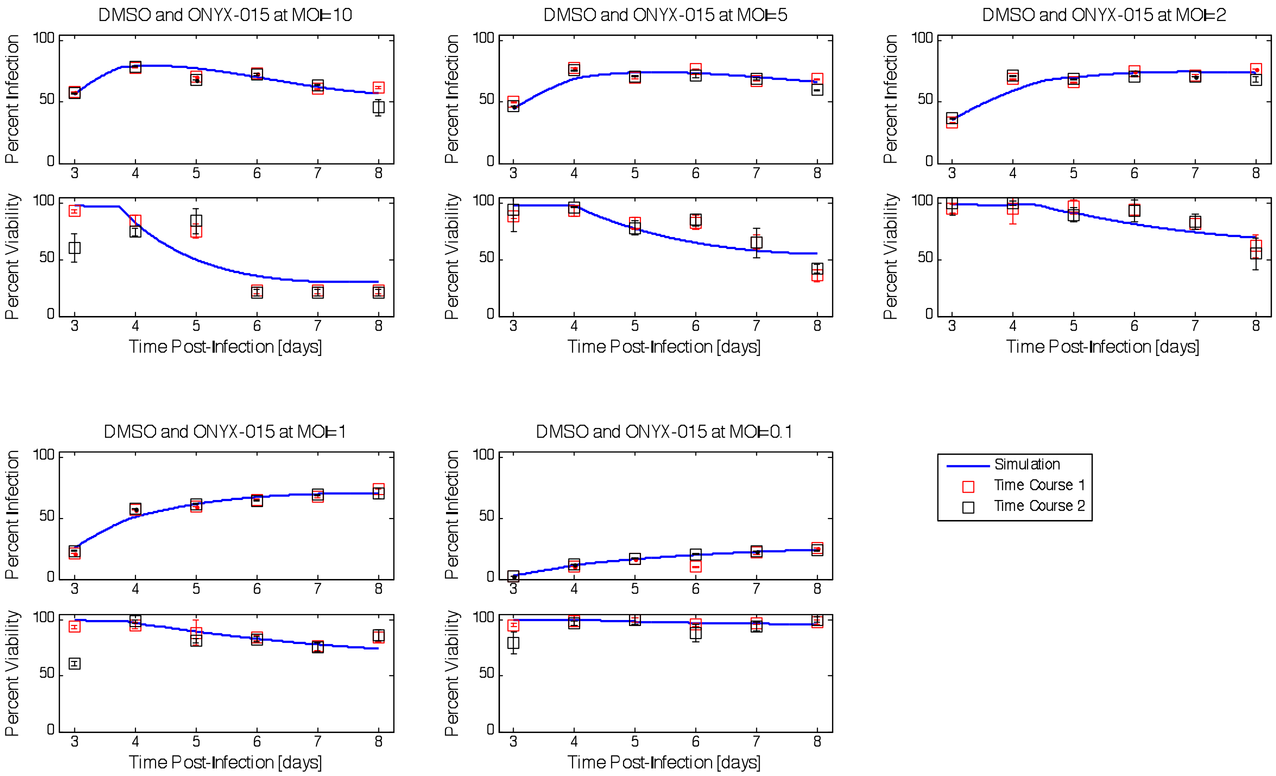

Figure S2: Infection and viability measurements upon DMSO treatment. Cells were treated with DMSO for 2 days. Following media change, cells were infected with GFP-expressing ONYX-015 (for measuring percent infection) and ONYX-015 (for quantifying cell viability) at MOIs 0.1, 1, 2, 5, & 10. Relative cell infection (upper subplots) and cell viability (lower subplots) were measured 1-6 days post-infection. Infection and viability data are shown as percent measurements with respect to the uninfected control (MOI=0). The blue solid lines reflect simulated dynamics, while the red and black square markers reflect experimental data. Error bars represent standard deviation of duplicate measurements.

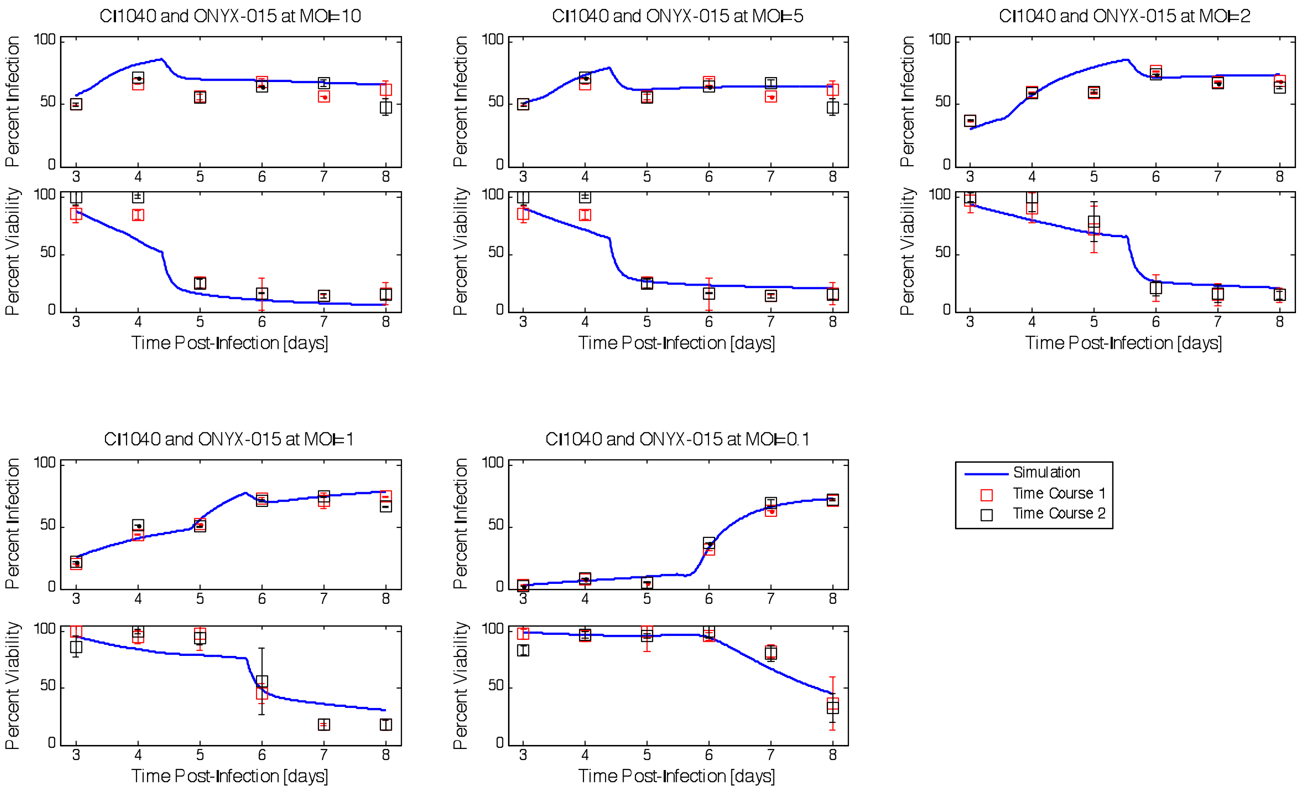

Figure S3: Infection and viability measurements upon CI1040 treatment. Cells were treated with CI1040 for 2 days. Following media change, cells were infected with GFP-expressing ONYX-015 (for measuring percent infection) and ONYX-015 (for quantifying cell viability) at MOIs 0.1, 1, 2, 5, & 10. Relative cell infection (upper subplots) and cell viability (lower subplots) were measured 1-6 days post-infection. Infection and viability data are shown as percent measurements with respect to the uninfected control (MOI=0). The blue solid lines reflect simulated dynamics, while the red and black square markers reflect experimental data.

Parameter Rate Const.	$t_d<t<t_w$ No infection	$t<t_d$ or $t_w<t$ No infection
σ; sat	1.59; 1.38e5	1.59; 1.38e5
arrest	3.43	0
release	0.36	0.36

Parameter Rate Const.	$t<t_d$ MOI = 0.1	$t<t_d$ MOI = 1	$t<t_d$ MOI = 2	$t<t_d$ MOI = 5	$t<t_d$ MOI = 10
β	.09	.46	.48	.63	1.00
$delay_\beta$	.68	.35	.04	.01	.14
δ	.23	.23	.24	.40	.78
$delay_\delta$	1.98	1.97	2.49	2.02	1.77

Parameter Rate Const.	$t<t_d$ MOI = 0.1	$t<t_d$ MOI = 1	$t<t_d$ MOI = 2	$t<t_d$ MOI = 5	$t<t_d$ MOI = 10
β_T	1.34	0.97	0.89	0.68	0.40
β_{TG1}	0.03	0.31	0.37	0.94	1.06
$delay_{\beta T}$	3.78	2.85	1.61	1.28	2.98
δ_T	1.04	5.65	6.45	6.62	4.04
$delay_{\delta T}$	0.49	3.75	3.55	2.39	2.15

Table S1: Resulting parameter values. In the first section of the table, we summarize proliferation-specific parameter values. In the second, we summarize infection and cell death specific parameter values for the control or DMSO case. In the last section of the table, we summarize infection and cell death parameter values for the treatment or CI1040 case. Note that there is a specific delay associated with infection and cell death for both DMSO and CI1040 treated cells.

Parameter Convergence

We assessed convergence of parameter estimation by evaluating the (W)SSE and resultant parameter values of 50 independent iterations of the optimization algorithm. If the algorithm returns consistent parameter estimates and consistent fitness (as denoted by the cost function), parameter identifiability issues are likely negligible since the optimization method is able to account for and estimate individual parameter values. Alternatively, if the algorithm returns consistent measures of fitness and variable parameter values, the model and algorithm may suffer from an inability to identify parameters independently. In the latter case, the cost function is relatively insensitive to corresponding parametric perturbations. A common cause of identifiability problems entails dependency among parameter values. A simple test involves assessing the correlation between resulting parameter values when such variance exists.

Among the proliferation parameters, *arrest* and *release* exhibited clear mutual dependence with a correlation coefficient of 0.993 (Figure S4). Their correlation was expected, however, since both parameters regulate the reversible G1 cell cycle arrest dynamics as a result of MEK-inhibitor. Similarly, *sigma* and *sat* exhibited a correlation coefficient of 0.821. Additional correlations were observed among certain infection and cell death kinetics in DMSO conditions. At higher MOI (i.e. 2, 5, and 10), the rates of infection and the rates of cell death are weakly correlated with coefficients between 0.201 and 0.353. The rates of infection or cell death are comparably correlated with their corresponding delay terms. Parameters that exhibited the strongest association involved the delay of infection and the rate of cell death (correlation coefficients between 0.604 and 0.796 across MOI). These observations are justified by the fact that cell death is an irreversible reaction that must take place once a cell is infected. At low MOI (i.e. 0.1 and 1), parametric correlations were inconsistent, which likely relates to the lack of experimental dynamics since few cells infect and lyse in low virus titer. CI1040 conditions also yielded inconsistent parameter correlations with respect to MOI. The one exception involved the rate of infection from the arrested

state, $\beta_{T\cdot G1}$, and the delay of infection from the treated proliferating state (correlation coefficients ranged between 0.493 and 0.802). The variability of parametric correlation in the CI1040 case is likely related to the greater complexity of the model structure. Here, cells may infect into the treated state, IC_T, via the proliferative state, C, or the arrested state, C_{G1}.

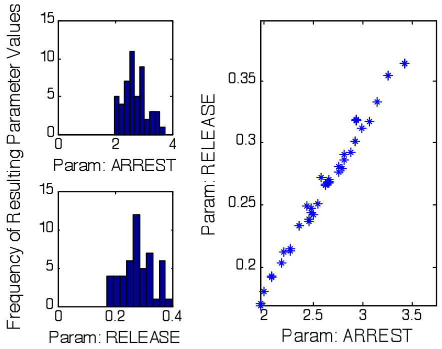

Figure S4: Identifying correlation between parameters *arrest* and *release*. Fifty iterations of independent parameter estimation demonstrated a correlation between parameters *arrest* and *release* with a correlation coefficient of 0.993. Such a strong association was expected since *arrest* and *release* govern the only reversible reaction in the system of equations: G1 cell cycle arrest. Subplots on the left depict the histogram of the 50 resulting parameter values while the plot on the right depicts their correlation.

The observed parametric correlations did not present adequate concern to motivate the derivation of a non-dimensional form of our nonlinear system of ODEs. Deliberate sequential parameter estimation required fitting a maximum of 5 parameters at any given time, limiting the uncertainty (or insensitivity) of the fitness function with respect to parameter values. To assess convergence, we computed each optimization algorithm 50 independent times. The resultant parameters exhibited negligible variation. Exceptions included both delay terms in the DMSO cases at low MOI, and parameters $\beta_{T\bullet G1}$ and $delay_{\delta T}$ in the CI1040 cases at low MOI. Given the limited experimental dynamics at MOIs of 0.1 and 1, we did not believe that reducing the number of fitted parameters would ensure greater precision or significantly affect the aims of our study.

Model Fitness

In using a maximum likelihood approach to estimate model parameters, we assumed that the residuals of our fitness function were normally distributed and zero mean centered. Therefore, model fitness is determined in part by whether this assumption holds. Figure S5 depicts the histogram of residuals resulting from each of the 50 independent iterations of parameter estimation. In the leftmost plot, residuals associated with *sigma*, *sat*, *arrest*, and *release* reflect a normal distribution at the 5% significance level with a mean of -0.90 (standard deviation of 6.94). In the middle plot, residuals associated with β, δ, and corresponding *delay* terms (DMSO treatment) reflect a normal distribution at the 5% significance level with a mean of 4.45 (standard deviation of 8.40). In the rightmost plot, residuals associated with β_T, $\beta_{T\cdot G1}$, δ_T, and corresponding *delay* terms (CI1040 treatment) reflect a normal distribution at the 5% significance level with a mean of 3.67 (standard deviation of 9.59). If all residuals are consolidated, they are normally distributed at the 5% significance level with a mean of 2.77 (standard deviation of 8.80).

Residuals, x, meet the criteria for normal distribution as a function of MATLAB's t-test provided a mean, m: 'ttest(x,m) performs a t-test of the null hypothesis that data in the vector x are a random sample from a normal distribution with mean m and unknown variance'. It is important to note that residuals are not zero-mean centered and fail the test if the mean is not specified. This deviation may be associated with initial conditions since the time interval that governs the ODE solver may not be consistent for each experimental measurement. In particular, the first time point (or residual) may bias the SSE metric since it is anchored to the first experimental measure and subject to experimental variation and initial conditions unaccounted for in the system of equations. Provided a non-zero mean of residuals, we cannot ensure that parameter estimates are optimal. However, given that normalized mean values (with respect to standard deviations) are minor (-0.13, 0.53, and 0.38 respectively) and that mean-centered residuals are normally distributed, a maximum likelihood approach to estimate model parameters is suitable.

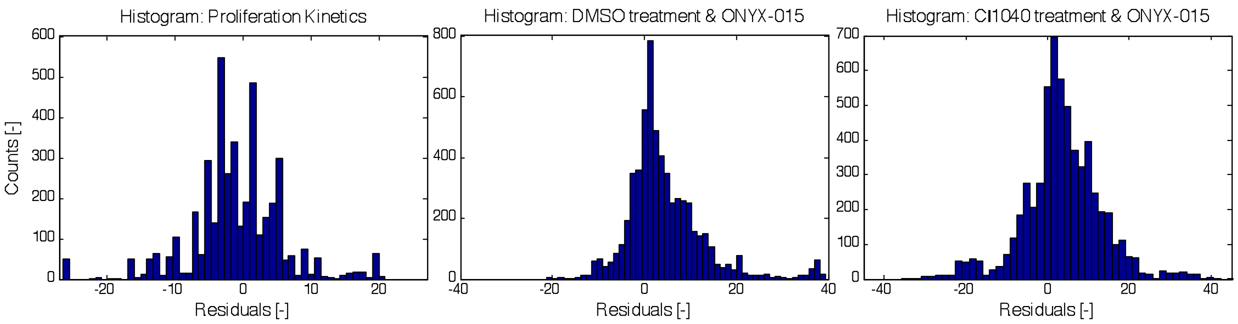

Figure S5: Histogram of residuals for parameter estimation. Fifty iterations of independent parameter estimation yield normally distributed residuals corresponding to proliferation, DMSO treated & infected, and CI1040 treated & infected conditions with mean values of -0.90, 4.45, and 3.67 respectively. Mean-centered residuals meet the criteria for normal distribution as evaluated by the default conditions of MATLAB's ttest function.

Interpolation Methods

By fitting a wide range of infection conditions we were able to establish a framework that allowed for interpolation of intermediate parameter values to account for a continuum of experimental and therapeutic possibilities. To assess the efficacy of MATLAB's 1D interpolation algorithms, we determined the total cost associated with interpolating known data points. More specifically, we omitted parameters corresponding to an MOI of 1, 2, or 5, interpolated the absent parameter value given the spectrum of known parameters, and calculated the SSE between the interpolated parameter simulation and the training data. The total SSE for these MOIs was determined for each relevant MATLAB interpolation method. Results demonstrated that cubic interpolation was most accurate for this system (Figure S6).

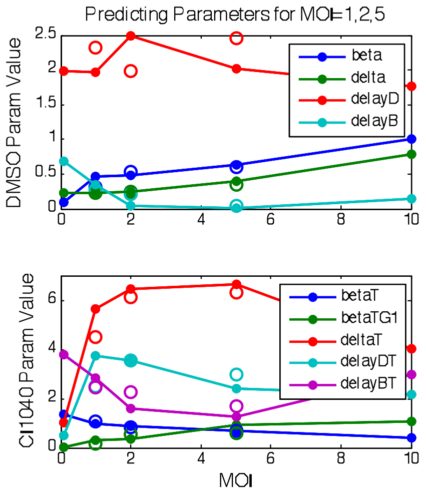

Figure S6: Cubic interpolation of intermediate parameter values. Solid-filled markers connected by solid lines reflect fitted parameter values that govern DMSO and CI1040 infection and cell death kinetics (upper and lower subplots, respectively). To assess the efficacy of interpolation methods, we interpolate known parameters associated with MOIs 1, 2, or 5, and determine the difference in SSE between the experimental data and simulations employing the fitted parameters (solid markers) versus their interpolated values (open markers). The open markers reflect the cubic interpolated values of parameters assuming the given MOI is individually omitted from the data.

Model Simulations

We interpolated resulting parameter values (Table S1) and used the model to predict the extent of cell death as a function of the time of CI1040 treatment initiation, time of ONYX-015 infection, and MOI. We employed an exhaustive search algorithm to simulate the effect of various treatment and infection protocols. This algorithm systematically evaluated every possible sequence combination of drug treatment and infection conditions (within a defined interval), with the exception of media change, t_w, which was set to occur 2 days after treatment. We varied CI1040 treatment initiation between days 0-3 and infection between days 0-7. The multiplicity of infection was also varied between 0.1 and 10 (Figure S7). We evaluated percent cell death on day 8 irrespective of the sequence protocol.

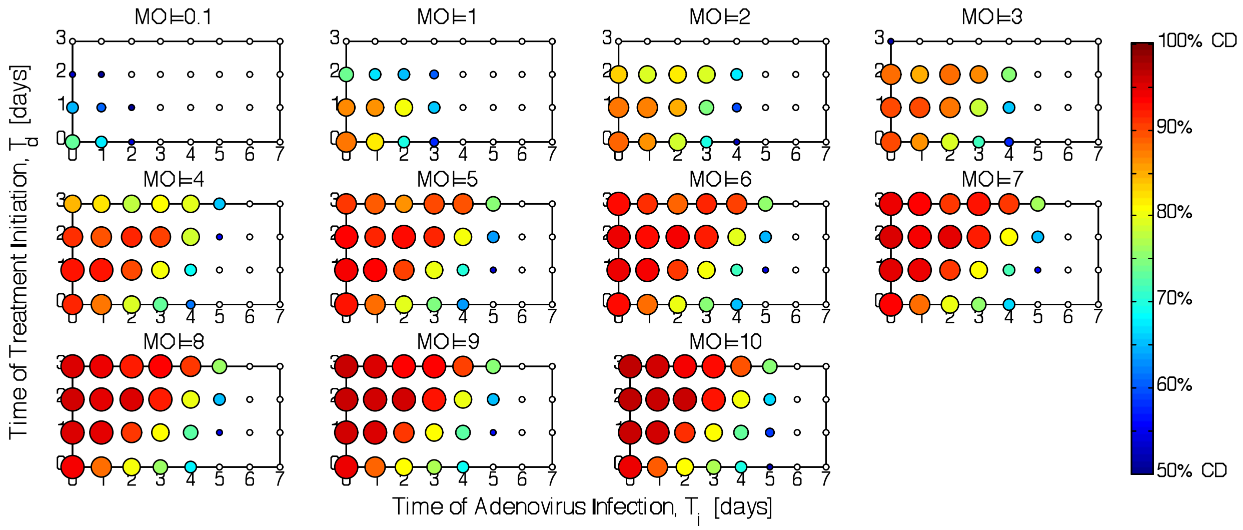

Figure S7: Simulated predictions of cell killing as a result of CI1040 treatment and ONYX-015 infection. Simulated percent cell death is evaluated on day 8 as a function of the timing of MEK-inhibitor treatment initiation, timing of infection, and multiplicity of infection (MOI). Each Cartesian coordinate reflects an independent simulation or treatment/infection protocol. The timing of ONYX-015 infection is varied on the x-axis; the timing of CI1040 treatment initiation is varied on the y-axis. CI1040 removal by media change occurs 2 days post treatment irrespective of the timing of infection. MOI is held constant in each subplot: 0.1, 1, 2, 3, 4, 5, 6, 7, 8, 9, and 10. Treatment and infection protocols that yield over 50% cell death are shown. Greater cell death (CD) is reflected by larger data points and an increasingly red color (see color bar). Empty data points depict protocols that fail to kill at least 50% of the cellular population.

MATLAB syntax for ODE model.

```
function output = fullODE(t,input,td,tw,ti,M)
C    = input(1);
C_G1 = input(2);
IC   = input(3);
IC_T = input(4);

P    = C + C_G1 + IC + IC_T;

sigma  = 1.59;
sat    = 1.38*10^5;
arrest = 3.43;
release= 0.36;

betaVal   = [0.09 0.46 0.48 0.63 1.00];
deltaVal  = [0.23 0.23 0.24 0.40 0.78];
delayDVal = [1.98 1.97 2.49 2.02 1.77];
delayBVal = [0.68 0.35 0.04 0.01 0.14];

betaTVal  = [1.34 0.97 0.89 0.68 0.40];
betaTG1Val= [0.03 0.31 0.37 0.94 1.06];
deltaTVal = [1.04 5.65 6.45 6.62 4.04];
delayDTVal= [0.49 3.75 3.55 2.39 2.15];
delayBTVal= [3.78 2.85 1.61 1.28 2.98];


% Interpolate Parameter values -------------------------------------------
MOI = [0.1 1 2 5 10];

beta   = interp1(MOI,betaVal,M,'cubic');
delta  = interp1(MOI,deltaVal,M,'cubic');
delayD = interp1(MOI,delayDVal,M,'cubic');
delayB = interp1(MOI,delayBVal,M,'cubic');

betaT   = interp1(MOI,betaTVal,M,'cubic');
betaTG1 = interp1(MOI,betaTG1Val,M,'cubic');
deltaT  = interp1(MOI,deltaTVal,M,'cubic');
delayDT = interp1(MOI,delayDTVal,M,'cubic');
delayBT = interp1(MOI,delayBTVal,M,'cubic');

% Proliferation ----------------------------------------------------------
if t<td || t>=tw
    arrest = 0;
end

% Infection --------------------------------------------------------------
if t<ti+delayB
    beta = 0;
end

% Viability --------------------------------------------------------------
if t<ti+delayD
    delta = 0;
end
```

```
if t>td % With Treatment -------------------------------------------------
    % Infection ---------------------------------------------------------
    if t<ti
        betaTG1 = 0;
    end
    if t<ti+delayBT
        betaT = 0;
    end

    % Viability ---------------------------------------------------------
    if t<max([ti+delayDT tw+delayDT])
        deltaT = 0;
    end

else % Without Treatment -------------------------------------------------
    betaT = 0;
    betaTG1 = 0;
    deltaT = 0;
end

% System of ODE ---------------------------------------------------------
dC    = sigma*C*(1-P/sat) - arrest*C + release*C_G1 - beta*C - betaT*C;
dC_G1 =                     arrest*C - release*C_G1 - betaTG1*C_G1;
dIC   = beta*C                 - delta*IC;
dIC_T = betaT*C + betaTG1*C_G1 - deltaT*IC_T;

output = [dC; dC_G1; dIC; dIC_T];

return
```

Minimum Information about a Flow Cytometry Experiment (MIFlowCyt) Outline

In this section, we outline how our flow cytometry experiments satisfy MIFlowCyt guidelines, as established by Lee et al.[1]. All FCS data files can be obtained by contacting Dr. Michael Korn.

Figure 1A: CAR Expression

1. **Experiment Overview**
   1.1. Purpose: The purpose of this experiment was to quantify CAR expression after HCT116 cells were treated with CI1040 or DMSO control.
   1.2. Keywords: HCT116 cells, CAR expression, CI1040.
   1.3. Experiments Variables: HCT116 cells were treated with CI1040, DMSO, or medium alone for 1 to 4 days and CAR expression was analyzed.
   1.4. Date: Flow cytometry analysis occurred on July 17, 2007.
   1.5. Results: Please refer to Results section in the manuscript.
   1.6. Quality Control Measurements:

Controls	Cells/Treatment	Primary Ab	Secondary Ab
Unstained	Treated and untreated cells	No	No
Primary Ab control	Treated and untreated cells	Yes	No
Secondary Ab control	Treated and untreated cells	No	Yes
Isotype control	Treated and untreated cells	IgG isotype control: Purified Mouse IgG1, isotype control. BD Bioscience Pharmigen (cat# 555746).	Yes
Cell viability control: sample stained with both antibody and PI nuclear stain.	Treated and untreated cells	Yes	Yes
Negative control	CHO minus cell*	Yes	Yes
Positive control	CHO plus cell*	Yes	Yes

*CHO cells: Chinese hamster ovary (CHO) cells were transfected with empty vector (CHO minus) or with cDNA constructs encoding human CAR (CHO plus). Dr. J. Bergelson (Division of Infectious Diseases, Children's Hospital of Philadelphia, PA, USA) kindly provided these cell lines.

2. **Flow Sample Details**
   2.1. Cell Line and Cell Culture: Please refer to the Materials and Methods section of the manuscript.
   2.2. Cell Line Characteristics: HCT cells are a human colorectal carcinoma cell line.
   2.3. Sample Treatment Description: Please refer to the Materials and Methods section of the manuscript.
   2.4. Fluorescence Detection: CAR signal was detected in FL1 using the standard filter configuration and PI signal was detected by FL3. Unstained and single fluorochrome stained cells were used to set up the machine.
3. **Instrument Details**
   3.1. Instrument Manufacturer: BD FACSCalibur Flow Cytometry (http://www.bdbiosciences.com/home/).
   3.2. Instrument Model: BD FACSCalibur Flow Cytometer System. Serial number: LCA S171-SNE3319.
   3.3. Flow Fluids: Medium Flow: 35µL/min.
   3.4. Lasers: Air-cooled argon-ion laser, 15 milliwatt, 488nm.
   3.5. Emission Detection:
   - *FL1 530/30*
   - *FL2 585/42*
   - *FL3 670 LP*
   - *FL4 661/16*

[1] MIFlowCyt: The minimum information about a flow cytometry experiment. Volume 73A, Issue 10, pages 926–930, October 2008.

- *SSC 488/10*
- *FSC 488/10*

4. **Data Analysis Details**
   4.2. Software: Data was analyzed with CellQuest Pro software (Becton, Dickinson and Company).
   4.3. Gating Description: After gating on the cell population in the FSC vs SSC plot, viable cells were identified by gating on SSC vs. FL3 (PI signal). CAR positive cells were detected in FL1 channel. Histogram markers were used and marker M1 was placed around the negative peak and marker M2 was placed to the right of M1 to designate positive events.
   4.4. Gate Statistics for the Controls: All test samples contain Primary Ab, Secondary Ab and PI. The results are shown in the Results section of the manuscript.

**Controls**	**Treatment**	**Primary Ab**	**Secondary Ab**	**PI**	**M1 (%gated)**	**M2 (%gated)**
Unstained	DMSO<br>CI1040	No	No	No	95<br>97	5<br>3
Primary Ab control	DMSO<br>CI1040	Yes	No	No	99<br>100	1<br>0
Secondary Ab control	DMSO<br>CI1040	No	Yes	No	99<br>99	1<br>1
Isotype control	DMSO<br>CI1040	Isotype control	Yes	No	99<br>99	1<br>1
Cell viability	DMSO<br>CI1040	No	No	Yes	97<br>98	1<br>1
Negative control	CHO<br>minus cell	No<br>Yes	No<br>Yes	No<br>No	98<br>97	2<br>3
Positive cell	CHO<br>plus cell	No<br>Yes	No<br>Yes	No<br>No	98<br>1	2<br>99

Figure 1C: Cell Cycle Profile

1. **Experiment Overview**
   1.1. Purpose: The purpose of this experiment was to analyze the cell cycle distribution after HCT116 cells were treated with CI1040 or DMSO control.
   1.2. Keywords: HCT116 cells, cell cycle distribution, CI1040.
   1.3. Experiments variables: HCT116 cells were treated with CI1040 or DMSO for 1 to 3 days and cell cycle distribution was analyzed.
   1.4. Date: Flow cytometry analysis occurred on August 07, 2009.
   1.5. Results: Please refer to Results section.
   1.6. Quality Control Measurements: Cells treated with CI1040 were also used as positive control as they arrest in G1.
2. **Flow Sample Details**
   2.1. Sample Treatment Description: Please refer to the Materials and Methods section in the manuscript.
   2.2. Fluorescence Detection: PI signal was detected in FL2W and FL2A using the standard filter configuration.
3. **Instrument Details**
   Same as information provided for Figure 1A.
4. **Data Analysis Details**
   4.1. Software: Data was analyzed with ModFit LT (Verity Software House).
   4.2. Gating Description: A gate was set around the single population in a FL2W vs. FL2A dot graph.

Figure 1D: GFP Expression

1. **Experiment Overview**
   - 1.1. Purpose: The purpose of this experiment is to quantify the GFP expression after HCT116 cells were treated with CI1040 or DMSO control and infected with ONYX/GFP.
   - 1.2. Keywords: HCT116 cells, CI1040, oncolytic adenovirus ONYX-015 expressing GFP.
   - 1.3. Experiment Variables: HCT116 cells were infected at 5 different MOIs of ONYX/GFP (0.1, 1, 2, 5, and 10).
   - 1.4. Date: Flow cytometry analysis occurred on July 10, 2009.
   - 1.5. Results: Please refer to Results section of the manuscript.
   - 1.6. Quality Control Measurements: HCT116 cells treated with CI1040 or DMSO control and not infected with ONYX/GFP were used negative samples.
2. **Flow Sample Details**
   - 2.1. Sample Treatment Description: Please refer to the Materials and Methods section of the manuscript.
   - 2.2. Fluorescence Detection: GFP was detected in FL1 using the standard filter configuration.
3. **Instrument Details**
   - 3.1. Instrument Manufacturer: Accuri Cytometers (http://www.accuricytometers.com).
   - 3.2. Instrument Model: Accuri C6 Flow Cytometer (http://www.accuricytometers.com/products/flow-cytometers/c6_flow_cytometer/). Serial number: LCAS-171 SN1915.
   - 3.3. Flow Cell: 200 micron ID quartz capillary.
     Flow Fluids: Medium flow at 35µL/min with 16µm core.
   - 3.4. Excitation: 488nm (rated at 20,000hr life).
     640nm (rated at 20,000hr life).
   - 3.5. Laser Profile: 15x75 microns.
   - 3.6. Emission Detection: 4 colors, user swappable optical filters. Standard set installed:
     - *FL1 530 / 30nm (FITC/GFP)*
     - *FL2 585 / 40nm (PE/PI)*
     - *FL3 >670nm LP (PE-Cy5, PE-Cy5.5, PerCP-Cy5.5, PE-Cy5, PE-Cy7)*
     - *FL4 675 / 25nm (APC)*
   - 3.7. Dynamic Range: The totally digital C6 Flow Cytometer has state-of-the-art electronics that make it possible to simultaneously collect over 16 million channels of digital data. This unparalleled dynamic range obviates the need for adjustable gain settings on the detectors and eliminating the need to set gain or voltage reduces.
4. **Data Analysis Details**
   - 4.1. Software: Data was analyzed with CFlow Software.
   - 4.2. Gating Description: Cells were gated on a FSC vs. SSC plot excluding debris. We used uninfected cells (GFP negative) to identify negatively-gated cells and all cells greater than the negatively-gated uninfected cells were considered GFP positive cells. Histogram markers were used and marker M1 was placed around the negative peak and marker M2 was placed to the right of M1 to designate positive events.
   - 4.3. Gate Statistics for Negative Cells. All test samples were treated with DMSO or CI1040 and infected with ONYX/GFP at different MOIs.

Treatment	M1 (% gated)	M2 (%gated)
DMSO uninfected	97	0
CI1040 uninfected	96	0

Figure 6A: Cell Cycle Profile on Density-Arrested Cells

1. **Experiment Overview**
   - 1.1. Purpose: The purpose of this experiment was to analyze the cell cycle distribution of HCT116 density-arrested cells.
   - 1.2. Keywords: HCT116 cells, cell cycle distribution, density-arrested cells.
   - 1.3. Experiments Variables: Cells were density-arrested by plating at 5×10^5 cells/cm^2 for 48h. Cells were subsequently re-plated at low density (1×10^5 cells/cm^2). Cell cycle distributions were analyzed at different times after the release of cells from arrest.

1.4. Date: Flow cytometry analysis occurred on April 24, 2009.
1.5. Results: Please refer to the Results section of the manuscript.
1.6. Quality Control Measurements: Cells treated with CI1040 for 24h were used as positive control.

2. **Flow Sample Details**
   2.1. Sample Treatment Description: Please refer to the Materials and Methods section of the manuscript.
   2.2. Fluorescence Detection: PI signal was detected in FL2W and FL2A using the standard filter configuration.
3. **Instruments Details**
   Same as information provided for Figure 1A.
4. **Data Analysis Details**
   4.1. Software: Data was analyzed with ModFit LT (Verity Software House).
   4.2. Gating Description: A gate was set around the single population in a FL2W vs. FL2A dot graph.
   4.3. Positive Control Sample:

Cell cycle phases	CI1040 treated cells
G1	87%
G2	4.6%
S	8.3%

Figure 6B: CAR Expression on Density-Arrested Cells

1. **Experiment Overview**
   1.1. Purpose: The purpose of the experiment is to quantify CAR expression after HCT116 cells were density arrested.
   1.2. Keywords: HCT116 cells, CAR expression, density-arrested cells.
   1.3. Experiments Variables: Cells were density-arrested by plating at 5×10^5 cells/cm^2 for 48h. Cells were subsequently re-plated at low density (1×10^5 cells/cm^2). CAR expression was analyzed at different times after release the cells from arrest.
   1.4. Date: Flow cytometry analysis occurred on November 8, 2011.
   1.5. Results: Please refer to Results section of the manuscript.
   1.6. Quality Control Measurements:

Controls	Primary Ab	Secondary Ab
Unstained	No	No
Primary Ab control	Yes	No
Secondary Ab control	No	Yes
Isotype control	IgG isotype control: Purified Mouse IgG1, isotype control. BD Bioscience Pharmigen (cat# 555746).	Yes
Cell viability control: sample stained with both antibody and PI nuclear stain.	Yes	Yes
Negative control CHO minus cell*	Yes	Yes
Positive control CHO plus cell*	Yes	Yes

*CHO cells: Chinese hamster ovary (CHO) cells were transfected with empty vector (CHO minus) or with cDNA constructs encoding human CAR (CHO plus). Dr. J. Bergelson kindly provided these cell lines.

2. **Flow Sample Details**
   2.1. Sample Treatment Description: Please refer to Materials and Methods section.
   2.2. Fluorescence Detection: CAR signal was detected in FL1 using the standard filter configuration and PI signal was detected by FL3. Unstained and single fluorochrome stained cells were used to set up the machine.

3. **Instruments Details**

   Same as information provided for Figure 1A.

4. **Data Analysis Details**
   4.1. Software: Data was analyzed with CellQuest Pro software (Becton, Dickinson and Company).
   4.2. Gating Description: After gating on the cell population in the FSC vs SSC plot, viable cells were identified by gating on SSC vs FL3. CAR positive cells were detected in FL1 channel. Histogram markers were used and marker M1 was placed around the negative peak and marker M2 was placed to the right of M1 to designate positive events.
   4.3. Gate Statistics for the Controls. All test samples contain Primary Ab, Secondary Ab and PI. The results are shown in the Results section of the manuscript.

Controls	Primary Ab	Secondary Ab	PI	M1 (%gated)	M2 (%gated)
Unstained	No	No	No	99	1
Primary Ab control	Yes	No	No	100	0
Secondary Ab control	No	Yes	No	99	1
Isotype control	Isotype control	Yes	No	99	1
Cell viability	No	No	Yes	100	1
Negative control<br>CHO minus	No<br>Yes	No<br>Yes	No<br>No	100<br>100	0<br>0
Positive cell<br>CHO plus	No<br>Yes	No<br>Yes	No<br>No	100<br>1	0<br>99

Figure 6D: Viability on Density-Arrested Cells

1. **Experiment Overview**
   1.1. Purpose: The purpose of this experiment is to analyze the viability of the density-arrested cells and infected with oncolytic adenoviruses.
   1.2. Keywords: HCT116 cells, density-arrested cells, viability.
   1.3. Experiments Variables: Cells were density-arrested by plating at $5{\times}10^5$ cells/cm^2 for 48 h and re-plated at low density ($1{\times}10^5$ cells/cm^2). Cell viability was analyzed at different times after release the cells from arrest and infected with different oncolytic adenovirus.
   1.4. Date: Flow cytometry analysis on June 1, 2009.
   1.5. Results: Please refer to Results section.
   1.6. Quality Control Measurements: Propidium iodide (PI, Sigma-Aldrich Co) was added just prior to acquisition to exclude dead cells from flow cytometric analysis. Uninfected HCT116 cells were used as negative samples.
2. **Flow Sample Details**
   2.1. Sample Treatment Description: Please refer to the Materials and Methods section of the manuscript.
   2.2. Fluorescence Detection: PI signal was detected in FL2 using the standard filter configuration.
3. **Instruments Details**

   Same as information provided for Figure 1D.

4. **Data Analysis details**
   4.1. Software: Data was analyzed with CFlow Software.
   4.2. Gating Description: Cells were gated on FSC vs. SSC plot excluding debris. PI was detected at FL2 and PI positive cells (dead cells) were excluded. The absolute numbers of viable cells were used for the analysis. Cell viability was expressed as percentage of the uninfected medium control (i.e. MOI=0).
